# Periodontal ligament stem cells as a promising therapeutic target for neural damage

**DOI:** 10.1186/s13287-022-02942-9

**Published:** 2022-06-21

**Authors:** Fariba Mohebichamkhorami, Roya Fattahi, Zahra Niknam, Morteza Aliashrafi, Sahar Khakpour Naeimi, Samira Gilanchi, Hakimeh Zali

**Affiliations:** 1 Department of Tissue Engineering and Applied Cell Sciences, School of Advanced Technologies in Medicine, Shahid Beheshti University of Medical Sciences, Tehran, Iran; 2 Faculty of Paramedical Sciences, Shahid Beheshti University of Medical Sciences, Tehran, Iran; 3 Department of Cognitive Neuroscience, Institute for Cognitive Science Studies, Tehran, Iran; 4 Department of Biology, Tehran Central Branch, Islamic Azad University, Tehran, Iran; 5 Medical Nanotechnology and Tissue Engineering Research Center, Shahid Beheshti University of Medical Sciences, Tehran, Iran

**Keywords:** Periodontal ligament stem cells, Neural regeneration, Neural differentiation, Neurotrophic factors, Apoptosis, Inflammation

## Abstract

**Background:**

The damaged neuronal cells of adult mammalian lack the regenerative ability to replace the neuronal connections. Periodontal ligament stem cells (PDLSCs) are the promising source for neuroregenerative applications that can improve the injured microenvironment of the damaged neural system. They provide neuronal progenitors and neurotrophic, anti-apoptotic and anti-inflammatory factors. In this study, we aimed to comprehensively explore the various neuronal differentiation potentials of PDLSCs for application in neural regeneration therapy.

**Main text:**

PDLSCs have superior potential to differentiate into various neural-like cells through a dedifferentiation stage followed by differentiation process without need for cell division. Diverse combination of nutritional factors can be used to induce the PDLSCs toward neural lineage. PDLSCs when coupled with biomaterials could have significant implications for neural tissue repair. PDLSCs can be a new clinical research target for Alzheimer's disease treatment, multiple sclerosis and cerebral ischemia. Moreover, PDLSCs have beneficial effects on retinal ganglion cell regeneration and photoreceptor survival. PDLSCs can be a great source for the repair of injured peripheral nerve through the expression of several neural growth factors and differentiation into Schwann cells.

**Conclusion:**

In conclusion, these cells are an appealing source for utilizing in clinical treatment of the neuropathological disorders. Although significant in vitro and in vivo investigations were carried out in order for neural differentiation evaluation of these cells into diverse types of neurons, more preclinical and clinical studies are needed to elucidate their therapeutic potential for neural diseases.

## Introduction

The central nervous system (CNS) and the peripheral nervous system (PNS) are parts of the nervous system. The brain and spinal cord make up the CNS, whereas cranial and spinal nerves, as well as their associated ganglia, constitute the PNS. The PNS has a built-in ability to regenerate and repair itself, whereas the CNS is essentially incapable of self-repair. Furthermore, depending on the characteristics and type of damage, the inherent regenerating ability is limited through injury itself [1].

In neural injuries, the damaged neural cells such as neurons and glial cells of adult mammalian lack the regenerative ability to replace the neuronal connections. This is due to the limited ability of neuronal progenitors to regenerate functional neuronal cells and inhibition of neural regeneration by the local injured microenvironment, especially in the glial scar [2].

One of the main formidable reasons for the limited success of pharmacotherapeutic strategies in neural damages is the microenvironment of the injury site with many molecular growth inhibitors that are hostile to any neuroregenerative therapy and function restoring of nerve fibers. It leads to the incapability of the damaged nerves to regrow and develop new synaptic connections. Targeting these inhibitors could be an efficient approach to overcome the permanent stopping of nerve growth [3].

The development of more precise therapies focused at specific molecular targets linked with a specific disease or injury of the nervous system has resulted from advances in neuroregenerative research. Due to several pathological injury processes and mechanisms, any neuroregenerative approach that focuses on just one of the events or mechanisms will not probably lead to a considerable therapeutic effect on neural injuries. The reasons for the limited therapeutic options are mainly because of both the extracellular and intracellular components of the nervous system that inhibits regeneration. To bridge the short-term requirements and revive immediate function of the nervous system, changes in plasticity and neuroregeneration firstly occur at the regional level. The lengthy and more permanent process of restoring function occurs at cellular level and promotes one or more of the restorative mechanisms which may improve neurological damages [4, 5].

Neuroregenerative medicine (NRM) is a growing field with the goal of neurogenesis, angiogenesis and synaptic plasticity [5] through replacement of lost cells and tissues and restoration of normal function [2]. Scientists are optimistic about the potentials of NRM to lead to providing novel approaches for the treatment of neural diseases and answer the ethical questions about their clinical applications [6].

NRM uses stem cells as a promising tool that make up for the scarcity of cell alternatives. Transplanted stem cells can improve the microenvironment in the injured site of the neural system and provide neuronal progenitors [7]; then, they help to slow or repair the deterioration related to degenerative or traumatic neural diseases and trigger a great effort in the field of preclinical and clinical neural research [8].

Mesenchymal stem cells (MSCs) have potential to integrate into host neuronal networks and renew functional neural connections. They can restore synaptic transmitter secretion, modulate the plasticity of damaged host tissues as well as release growth and neurotrophic factors with ability to promote cell survival [9]. In addition, MSCs have been found to diminish inflammation in vivo by suppressing pro-inflammatory cytokines and increasing anti-inflammatory cytokines and antigen-specific T-regulatory cells [10]. Researchers suggested that MSCs can cross the blood–brain barrier (BBB) [11], and this ability is the main reason for the treatment potential in neural diseases like cerebral ischemic diseases or spinal cord injuries [12, 13].

The oral cavity as an available source of MSCs includes two kinds of cells. Nondental oral MSCs which comprise periodontal ligament stem cells (PDLSCs), gingival MSCs (GMSCs), and dental follicle stem cells (DFSC) and the dental MSCs which consist of stem cells from apical papilla (SCAP), dental pulp stem cells (DPSCs) and stem cells from exfoliated deciduous teeth (SHED) [7].

Oral stem cells are rather accessible and show broad differentiation potential and high plasticity; hence, they can make autologous cell transplantation possible [8]. Moreover, they have advantages such as a higher proliferation rate and potential of immunosuppression [14, 15]; therefore, they are an excellent cell source in order for allogeneic transplantation.

They originate from cranial neural crest-derived ectomesenchymal cells (CNCCs); thus, they are capable of differentiation into neural cells in order for the reconstruction of central nervous system tissues. These cells express neural progenitors markers, including nestin, Pax6, Tuj1 and p75/NGFR, and have a more favorable neurotrophic secretome [16].

## Periodontal ligament-derived MSCs

PDL is the connective tissue that connects the root of the tooth to the alveolar bone socket, and it is derived from embryonic CNCCs. It includes bone and cementum cells and a mix of fibroblasts, mesenchymal and undifferentiated cells, all of which are seated on a hydrated fibrillar extracellular substance rich in collagen [17]. Human periodontal ligament stem cells (hPDLSCs) derived from PDL. PDLSCs are an ethical and accessible source of cells. These are an ample source of undifferentiated cells with a doubling rate of 22 h from periodontal ligament (PDL) with low rate of immunogenicity and can be transplanted without need for pre-differentiation, and they have the potential for the off-the-shelf product [2].

**Fig. 1:**
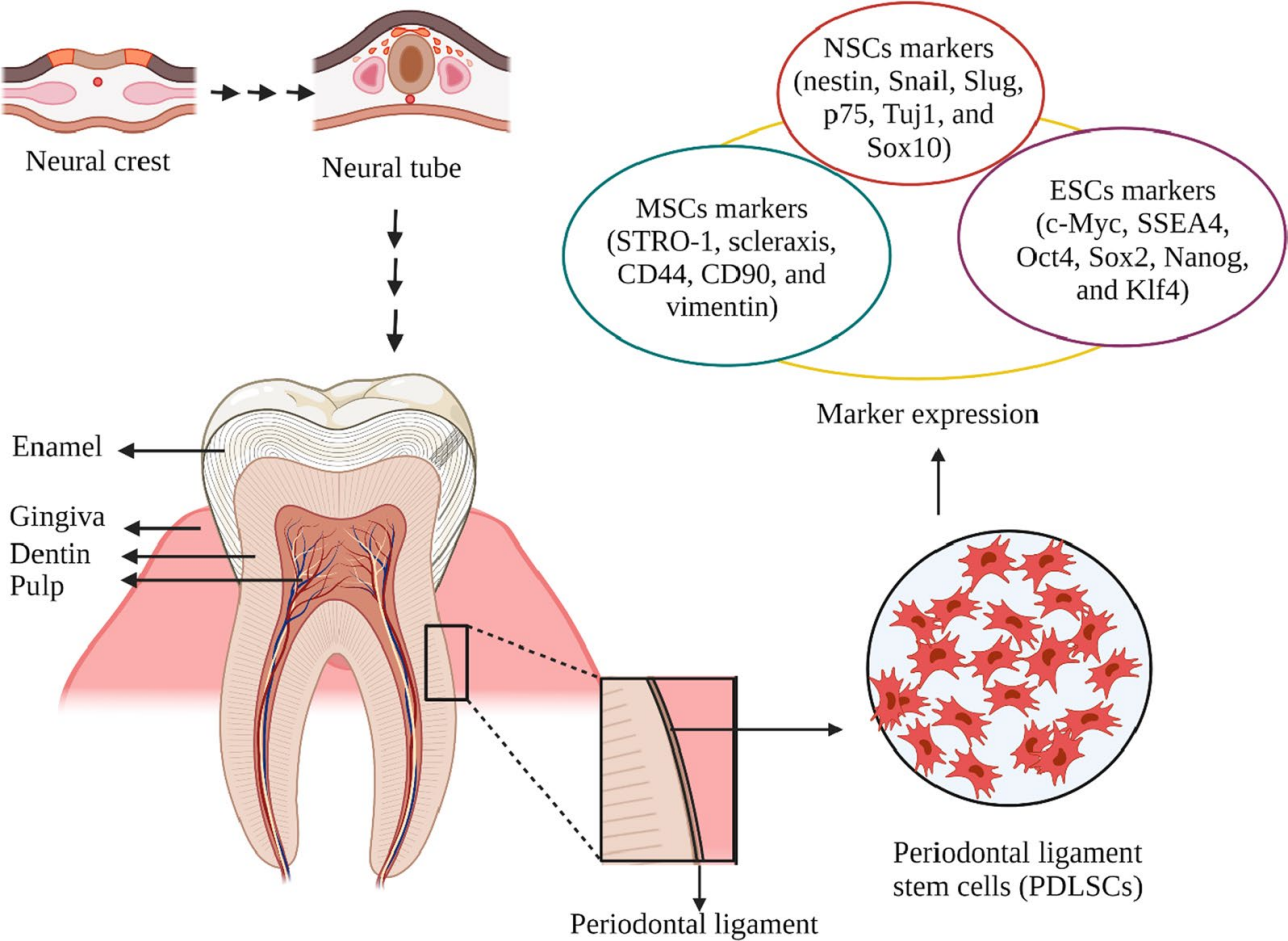
Schematic illustration of periodontal ligament stem cells characteristics. Neural stem cells (NSCs). Mesenchymal stem cells (MSCs). Embryonic stem cells (ESCs). Created with BioRender.com

Periodontal ligament stem cells (PDLSCs) contain 95% of mesenchymal stem cells and 5% of neural crest stem cells [18]. These cells have the ability to grow clonally and express MSCs markers (vimentin, STRO-1, CD90, scleraxis, CD44), markers of embryonic stem cells (c-Myc, SSEA4, Klf4, Oct4, Nanog and Sox2) and neural crest cells markers (Sox10, nestin, Snail, Slug, Sox10 p75 and Tuj1). These markers are considered as hallmarks of multipotency that highlight the differentiation capacity of PDLCs into various cell types, such as chondrogenic, cardiomyogenic, osteogenic and neurogenic lineages [19–21]. (Fig. 1).

PDLSCs possess cell replacement potential without tumorigenicity in cultured PDL cells. PDLSCs can exert an immunomodulatory effect via inflammatory cell recruitment [22, 23]. Different spectra of neurotrophic factors secretion were indicated by human PDLSCs such as BDNF, NGF, CNTF, bFGF, NT-3, VEGFA, IGF-1, and GDNF. Transplantation of different source of hPDLSCs like autologous and allogeneic sources can be promising paracrine-mediated and cell-free therapy for neural damage. The cell surface markers and differentiation potentiality of PDLSCs are similar to that of BMSCs and DPSCs [24], although it is demonstrated that the pluripotency and regenerative capacity of PDLSCs decrease with age [25].

### Isolation and characterization of PDLSCs

#### PDLSCs isolation

PDLSCs isolation was described for the first time by Seo BM [26] and Trubiani [27]. These stem cells can be isolated with different techniques and expanded in a xeno‐free medium with no alteration in morphological properties, their normal karyotype, and markers related to pluripotency. PDLSCs can be easily harvested from periodontal tissue during the noninvasive procedure [28, 29]. Different methods that are described to this date have some technical problems, but a simplified and highly replicable method is required [30].

Periodontal ligament biopsies can be obtained from root planning and dental scaling procedure. The first and second premolar teeth are suitable for the purpose of PDLSCs isolation. Teeth must be healthy with no sign of caries, periapical lesion, periodontal disease or gingivitis. Teeth are transferred in the cell culture medium supplemented with 1% antibiotic at 4 °C. In all seeding techniques, complete Dulbecco’s modified Eagle medium (DMEM) (supplemented with L-glutamine, 1% antibiotic, 10% of FBS) is used in standard culture conditions (37 °C with 5% CO2). The cells are isolated with two different methods through enzymatic digestion with type 1 collagenase or trypsin/EDTA and mechanical disaggregation and explant culture [30].

For enzymatic digestion, tissue is digested during 1 h at 37 °C in 500 µl type I collagenase at a concentration of 0.5 mg/ml or 500 µl of 0.25% trypsin with 0.1% EDTA [31]. At 10-min intervals, the sample is shaken every 30 s. The enzyme is inactivated with complete DMEM. Filtration of suspension is carried out with a 70-µm-diameter mesh to prepare a single-cell suspension, and centrifugation is performed at 1200 RPM/10 m. The supernatant is removed, and sedimented tissue is resuspended in complete DMEM. 300–500 µL of suspension is seeded in 6-well plates, and the culture medium is changed every 3 days. The advantages of this method are higher vitality of samples during transportation with DMEM, and also, conducted rinses in this method are efficient. Disadvantages of this technique are sample filtering and prolonged time for enzymatic digestion [30, 32].

For explant, cultured tissue fragments of approximately 1 mm diameter are minced and seeded on a culture plate or flasks with a minimum amount of complete DMEM. Explants are placed in the incubator under standard conditions, and it takes 6–8 h to adhere to the culture plate. After cell attachment, 500 µL of complete cell culture media is added. After 72 h, to discard floating tissue fragments, medium is changed. The advantage of this technique is that the use of explants provides high expectancy to access primary tissue; the disadvantage is very prolonged time for explant adhesion and washes with deficient time [30, 33].

#### PDLSCs characterization

##### Cell migration assessment

After the cells reached confluency, a lesion that causes continuity loss between PDLSCs is performed along the culture. The culture is monitored with photographs taken at different time points (12, 24, 48 and 72 h). After 12 h of lesion onset, cell migration toward the scratched area can be observed. Cell numbers increase after 24 h, and at 48 h after lesion onset, cells are in semi-confluence, and after 72 h, cells totally can cover the area [34].

##### Flow cytometric analysis for cell surface markers

Multi-parametric flow cytometry can analyze the expression of cell surface markers of PDLSCs. PDLSCs from passage 4 are used for this experiment. Trypsinized cells are counted using a hemocytometer, adjusted to the concentration of 0.7 − 10^6^ cells in 700 ml PBS, and equally divided into seven separate flow tubes. Cells (1 × 10^6^) are incubated for 45 min at 4 °C with the specific antibodies conjugated with fluorescein isothiocyanate (FITC). Cells are stained using antibodies against CD105, CD73, CD166, CD90, CD34, CD45, CD13, CD29, CD44, CD146, HLA-DR, OCT3/4, Sox2 and Nestin, SSEA4 intracellular antigens, p75, SOX10, CD49, SLACS and neural crest-related markers to study the cell surface characteristics [32, 35, 36]. The CLPP, NQO1, SCOT1, DDAH1 and a new isoform of TBB5 proteins that are related to the cell cycle regulation and homing, stress reaction and detoxification can be studied in PDLSCs [37]. After incubation with each antibody, the cells are washed adequately and characterized with a flow cytometer. PDLSCs are negative for the hematopoietic markers, which are essential for defining mesenchymal cells.

##### Trilineage differentiation of the PDLSCs

PDLSCs with upon 80% confluency are trypsinized and counted. A total 5.1 − 10^5^ cells are seeded into wells (4.3 − 10^3^ cells per cm^2^). After cells reached 80% confluency, media is changed from complete culture media to specific trilineage differentiation media (adipogenic, osteogenic and chondrogenic media). The differentiation media is changed every 4 days, and the cells are fixed, rinsed and stained on day 28; then, the adipogenic, osteogenic and chondrogenic differentiation potential of PDLSCs can be analyzed by staining with Oil Red O, Alizarin Red and Alcian blue stains, respectively. The cells are left for 30 min and 50 min at room temperature for staining with Alizarin Red/Alcian blue and Oil Red O, respectively. Following staining, the wells are washed with water, and water is added to each well to allow for visual inspection during imaging and prevent the cells from drying [32].

##### Gene analysis with reverse transcriptase-polymerase chain reaction (RT-PCR)

The total RNA is extracted from the isolated PDLSCs and used for the synthesis of cDNAs through RT-PCR. The expression profile of pluripotency markers (including Oct4A, Sox2 and NANOG) is evaluated and read by gel electrophoresis. The GAPDH gene is used as the control in the gel. The expression of GAPDH and Sox2 in PDLSCs is more intense than that of Oct4A and NANOG [32, 34].

#### Neural differentiation potential of PDLSCs

Since PDLSCs have the same embryologic origin of the neural crest with many mature neural cell types, they are less lineage-divergent to neural cells compared to many other stem cells types; therefore, these options make PDLSCs promising for neuroregenerative applications [2, 7, 38].

**Fig. 2:**
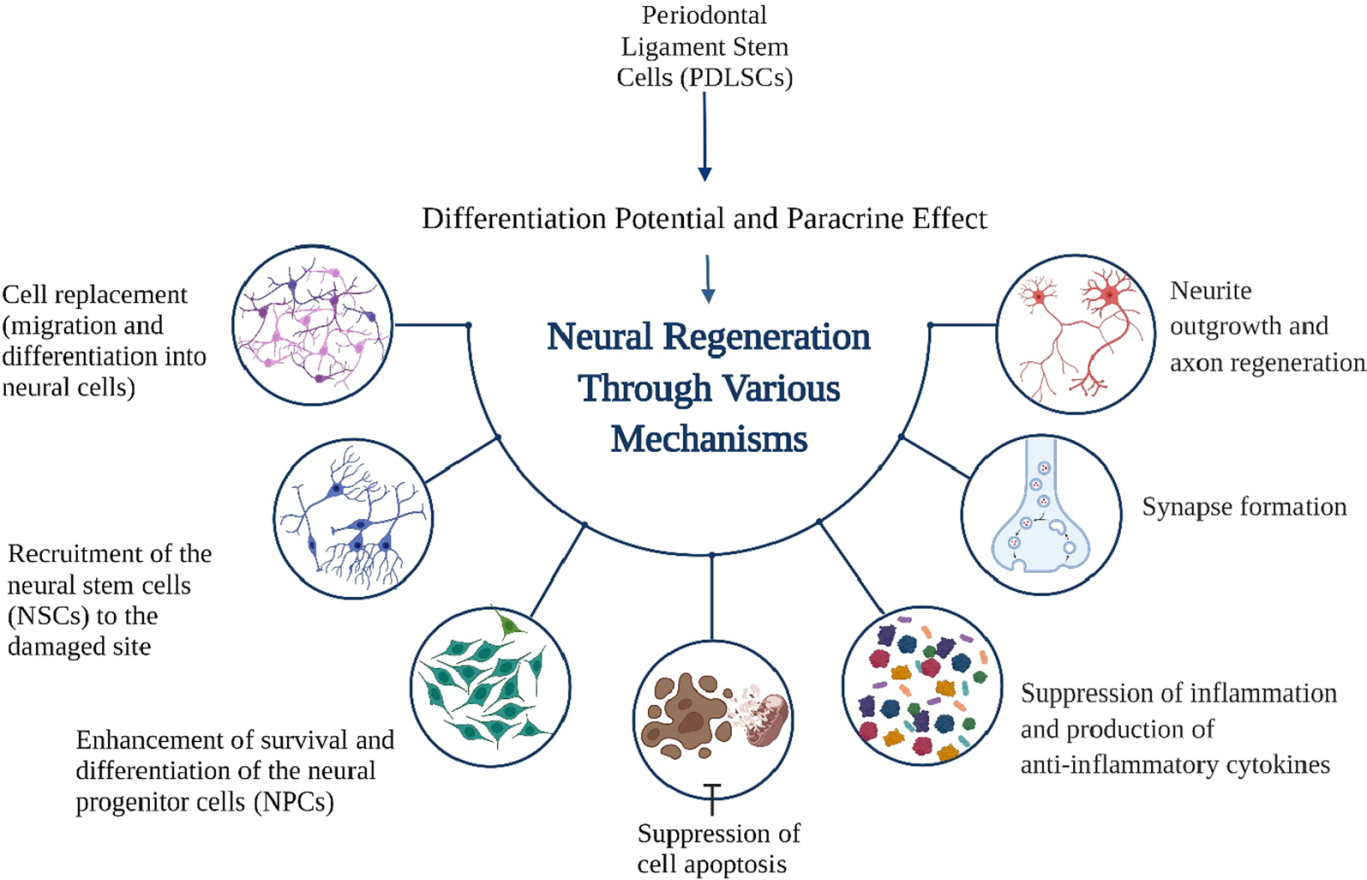
Mechanisms of neural regeneration by periodontal ligament stem cells. Created with BioRender.com

PDLSCs mediated neural regeneration through different mechanisms (Fig. 2) including migrating to the defect site and replacing the damaged cells [39] or recruiting the endogenous neural stem cells in injured site [40]. Moreover, they exert paracrine effect via increased expression of neurotrophic factors under the induction of neural injury environment and lead to stimulation of the neural progenitor cells to survive and differentiate [33, 41, 42]. They promote axon regeneration and synapse formation. Also, they can suppress cell apoptosis which is the major reason of neuronal loss in spinal cord injury (SCI) and Alzheimer’s disease (AD) [33, 40]. In addition, they inhibit the release of TNFα and upregulate the expression level of anti‐inflammatory cytokines [43, 44].

In this study, various neuronal differentiation potentials of PDLSCs for application in neural regeneration therapy are summarized. An additional file shows this in more detail (see Table 1).

**Table 1:** Potential of periodontal ligament stem cells in neural regeneration

Cells	Induction	Effects	Delivery method	Target molecules	Type of study	Ref
PDLSCs	With bFGF + EGF	Differentiated to neuronal and glial cells patch clamp showed outward and inward currents	–	βIII-tubulin↑ nestin↑ GFAP^+^ synaptophysin ^+^ SOX1↑ NEFM↑ Noggin↓	In vitro	[2]
PDLSCs	With bFGF and EGF	Differentiated to neural crest stem cells, glutamate‐induced calcium responses	–	βIII‐tubulin↑ NeuN↑ neurofilament↑ S100↑ neuron‐specific enolase↑ GFAP↑ miR‐132↑ ZEB2↓	In vitro	[51]
Xeno‐free hPDLSCs		Differentiated to a very small and rounded cell body with thin neurite‐like projections	–		In vitro	[13, 64]
PDLSCs + GMSCs	With NGF	Several islands of dense structures, positive for neurogenic specific markers, candidates for nerve tissue engineering	Encapsulated in alginate/hyaluronic acid hydrogel	βIII-tubulin↑ GFAP^+^ VAMP2^+^	In vitro	[57]
PDLSCs	Co-culturing PDLSCs with OA-induced SH-SY5Y cells	Recovery of cytoskeleton structure, cell shape and viability ↓apoptosis of okadaic acid‐induced SH‐SY5Y cells pTau	–	↓apoptosis related molecules	In vitro	[59]
PDLSCs		↓Inflammation and demyelination in the spinal cord in multiple sclerosis (MS) in animal model	Single-dose intravenous injection to the tail vein	↓inflammation and demyelination molecules, neurotrophic factors↑	In vivo	[60]
DPSCs and PDLSCs		Migrate toward infracted areas and differentiate into neuron-like cells, in vivo in rat model of cerebral ischemia	Direct administration	↓Notch pathway molecules ↑Wnt signaling pathway molecules Wnt3a↑ enolase ↑ tubulin↑ Thy-1↑ Jagged-1↓	In vivo	[61]
PDLSC-CM	PDLSCs obtained from RR-MS patients	Reduce inflammatory damage in the animal model of MS (in EAE mice)	Systemic administration	TGF‐β^+^IL‐10^+^	In vivo	[62]
PDLCS-CM	under hypoxic conditions	Pathology‐independent ability of PDLSCs niche ↑functionality of the PI3K/Akt/mTOR axis modulate markers of oxidative stress, autophagy and apoptosis	Injection of vesicles or conditioned medium to MS animal model	↓pro-inflammatory and ↑anti-inflammatory cytokines beclin‐1↑ LC3↑ Interferon‐γ↓ IL‐17↓BDNF↑	In vitro in vivo	[62, 65, 66]
hPDLSCs-CM		Enhanced level of NFκB and TLR4 and decreased amount of IκB-α in lipopolysaccharide-stimulated NSC34 mouse motoneurons	–	↑NFκB and TLR4 ↓IκB-α	In vitro	[67]
PDLSCs	PDLSCs are in contact with retinal ganglion cells (RGCs) in retinal explant culture	Neuroprotective effect and enhanced neurite regeneration in retinal tissue without macrophage recruitment	–	BDNF↑	In vitro	[68, 69]
PDLSCs		Electrically functional RGC survival and axonal regeneration in vivo in rat model of optic nerve injury	Intravitreal transplantation of PDLSCs		In vivo	[70]
PDLSCs		Differentiated into RGCs expression of ATOH7, POU4F2, β-III tubulin, MAP2, TAU, NEUROD1 and SIX3 formed synapses spontaneous electrical activities glutamate-induced calcium responses	–	VEGF↑ PTEN↑	In vitro	[69]
PDLSCs	Induction through chemically inhibiting Wnt and BMP signaling on Matrigel-coated surface	Differentiated into photoreceptor rosette-like outgrowth and excitatory glutamate response (Nrl^+^ rhodopsin^+^ Pax6^+^)	–		In vitro	[68, 71]
PDLSCs	Erk1/2 signaling	Differentiated into Schwann cells	–	P75↑ S100↑ GFAP↑ P0↑ krox-20↑ Oct-6↑	In vitro	[17, 77, 78]
PDLSCs	indirect co-culturing of heterogenous Schwann cells and PDLSCs (allogenic neurotrophic factors released by Schwann cells)	Presenting Schwann cell phenotype	–		In vitro	[75]
PDLSCs	sandblasted and acid-etched (SA) titanium surface	Schwann-like cells highest expression of SC markers and proteins on the SA titanium surface	–		In vitro	[79]
CM of the SCAPs, PDLSCs, and DPSCs	Induction of the cells with mixture of growth factors, Induction of SH-SY5Y cells with CM of the stimulated cells (in vitro)	BDNF↑ GDNF↑ in injured area, enhanced neurite outgrowth in SH-SY5Y cells in vitro, reduced the expression of caspase-3, higher level of neuronal markers in PDLSCs and SCAP, in rat sciatic nerve injury model	Transplantation of seeded fibrin glue conduits. Every fibrin glue conduit seeded with one of the stimulated cells and rat Schwann cells (rSC)	BDNF↑ GDNF↑ caspase-3↓	In vivo	[41, 80] [82]
PDLSCs		Recovery of sensory function ↑myelinated axons and retrograde labeled sensory neurons crush-injured left mental nerve in rats	Injected into the crush-injured left mental nerve	↑NGF receptor	In vivo	[80, 81]

Widera et al. [45] reported that PDLSCs are capable of differentiation into neural cells and glial cell lineages [45]. PDLSCs have potential to differentiate into neurofilament positive neuron-like cells, cyclic nucleotide phosphodiesterase-positive oligodendrocyte-like cells, and astrocyte-like cells with positive GFAP [46]. PDLSCs can successfully differentiate into neural-like cells in 8–16 days [2].

The sequence of the maturation of the neural-like cells from stem cells does not always need cell division. In vitro neurogenesis from hPDLSCs does not result in cell proliferation; it shows micronuclei movement and transient lobulation of cell nuclei, which is strikingly comparable to primary neuronal cultures and neurogenic niches in adult mouse brain [47].

Furthermore, there is a theory that MSCs transdifferentiate directly into neural cells, but this interpretation has been questioned. The fundamental criticism against this method is that the MSCs quickly adopt the morphologies of neuron-like cells by retracting their cytoplasm rather than actively extending neurites. Time-lapse microscopy was used to examine the order of biological cascades during neural differentiation processes of hPDLSCs, human bone marrow mesenchymal stem cells (hBMSCs) and hDPSCs. It was demonstrated that these cells shrink substantially and via extension of neurites they change their shape to neuron-like cells; the important point is that a dedifferentiation stage occurs prior to differentiation into neural phenotypes [48].

It is studied that growth factors have positive effects on the neural differentiation of PDLSCs. The nutritional factors like basic fibroblast growth factor (bFGF), nerve growth factor (NGF), L-glutamine, insulin-like growth factor-1 (IGF-1) and epidermal growth factor (EGF) are leading to induction of PDLSCs differentiation into neuronal lineage [49]. NGF is an effective inducing factor. Preconditioning of PDLSCs with dimethyl sulfoxide leads to better results [50].

Through using a combination of bFGF and EGF, PDLSCs can be induced into neural-like cells. Morphological changes to both neuronal and glial phenotypes were evident after this neuro-induction treatment and a significant increase was detected in b-tubulin III and nestin expressions, as well as positive staining of GFAP. Moreover, neural connections have been demonstrated with positive staining of synaptophysin in immunohistochemical analysis. The whole-cell patch clamping verified the outward and inward currents via voltage-gated sodium channels. In this process, PDLSCs differentiated into the neural cells at different rates because CD133 (human stem cell marker) did not show statistically significant change and SOX1 and NEFM noticeably but not statistically increased compared to control. Noggin that commonly has a function in neural development significantly decreased most likely due to increasing in matured cells [2].

Neural crest stem cells (NCSCs) differentiated from hPDLSCs were induced through differentiation medium containing bFGF and EGF for 24 days. The cells showed glutamate‐induced calcium responses and time-dependent increase in markers of neuronal and glial cells including βIII‐tubulin, NeuN, neurofilament, S100, neuron‐specific enolase and GFAP. The process of neuronal differentiation of human NCSCs is aided by miRNA regulation. After differentiation, sixty human miRNAs have been upregulated and nineteen of them downregulated. miR‐132 overexpression in NCSCs led to the downregulation of ZEB2 as a negative regulator of neuronal differentiation. The gene ontology analyses demonstrated that target genes of miR-132 are often involved in the neural differentiation processes such as axon guidance and neuron projection; therefore, miR‐132 can help to neural differentiation of NCSCs [51].

Xeno‐free hPDLSCs due to neural crest origin express neural markers as nestin and GAP‐43 spontaneously. GAP-43 is present in the periodontal Ruffini endings that play a substantial role in nerve repair and development [52]. GAP‐43 involves in mechanisms control branching and pathfinding of the glial cells [53, 54]. The PKCα/GAP‐43 nuclear signaling pathway controls neural differentiation of PDLSCs. A cytoskeleton rearrangement occurs during the process and shows a rounded and small cell body with thin projections like neurites [55, 56].

PDLSCs and GMSCs were encapsulated in alginate/hyaluronic acid hydrogel with sustained releasing of NGF. The encapsulated PDLSCs exhibited a higher level of neural genes expressions such as βIII-tubulin and GFAP and more numbers of cell colonies compared to GMSCs; however, both cells exhibited higher β -tubulin III expression than bone marrow MSCs. In all treated groups, VAMP2 (a synaptic protein) was positive. It was demonstrated that the elasticity of the hydrogel and biochemical microenvironment can impact on the rate of differentiation and proliferation of the encapsulated cells. The lowest level of elasticity led to the highest level of β-tubulin III expression. Moreover, the inductive signals can show the road of differentiation process. Seeded hydrogels were subcutaneously implanted in immunocompromised mice. Immunofluorescence and histochemical staining after 4 weeks of implantation showed several islands of dense structures and positive for neurogenic specific markers inside the cell-seeded hydrogels [57].

## Central nervous system treatment

**Fig. 3:**
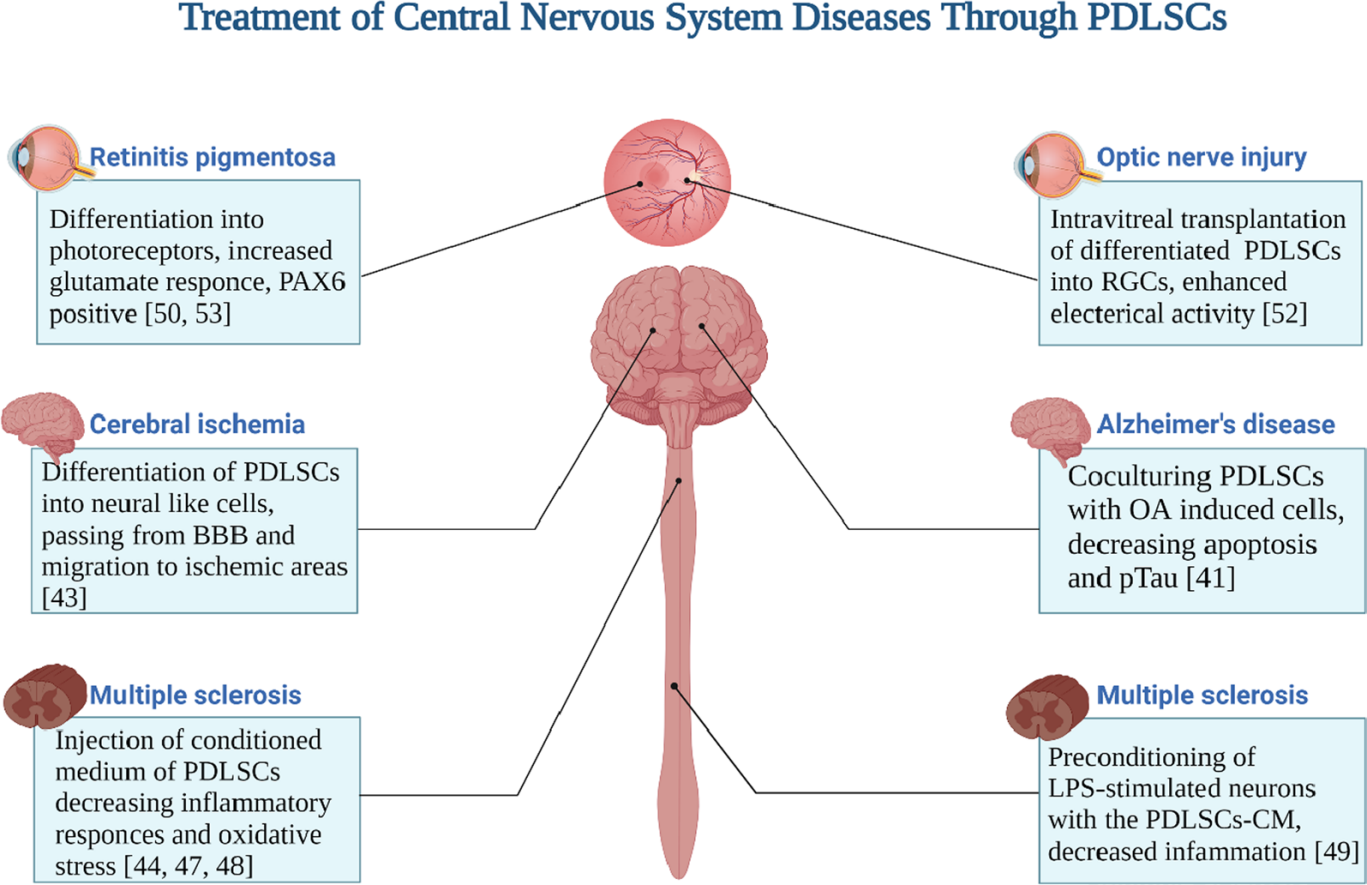
Treatment of disease in central nervous system through periodontal ligament stem cells. Retinal ganglion cells (RGCs). Blood brain barrier (BBB). Okadaic acid (OA). Lipopolysaccharide (LPS). Created with BioRender.com

The regenerative potential of PDLSCs in CNS diseases has been proven [51]. PDLSC engraftment and differentiation into the neural cells in adult mouse brain provided strong evidence for the utilization of these cells in order for cell therapy of neurodegenerative diseases [58]. (Fig. 3).

Effects of PDLSCs on the morphology and function of okadaic acid (OA)-induced SH-SY5Y cells as models of AD were investigated in vitro. PDLSCs are capable of regenerating tissues that have been damaged by AD characterized by deposited tau protein (pTau). 24-h co-culturing PDLSCs with OA-induced SH-SY5Y cells confirmed recovery of cytoskeleton structure and cell shape as well as cell viability compared to control. Apoptosis levels in SH-SY5Y cells induced by OA were decreased, and pTau expression was reduced [59]. Therefore, PDLSCs can be a new clinical research target for the AD treatment. Further studies about the effect of PDLSCs on the molecular and behavioral changes in AD are required.

Single-dose intravenous injection of 10^6^ hPDLSCs per 150 μl into the tail vein of the multiple sclerosis (MS) animal model at the beginning of the disease can decrease the signs of inflammation and demyelination in the spinal cord of the animals. It is due to the suppression of inflammatory mediators and the increased production of neurotrophic factors [60].

The DPSCs’ and PDLSCs’ differentiation into neuron-like cells was performed, and their therapeutic effects were investigated after administration in rat model of cerebral ischemia. Results exhibited that the expressions of Wnt3a, enolase and tubulin were noticeably enhanced and the expression of Jagged-1 was markedly decreased after neurogenic induction in both groups of cells but with more pronounced change in PDLSCs. PDLSCs showed a higher differentiation rate and expression of the Thy-1 (a neuronal differentiation marker). The Notch inhibition and Wnt signaling activation were detected in this process; therefore, these pathways have a regulatory role in the neural differentiation of the cells. In immunofluorescence studies, the spatial distribution and survival of labeled cells in the brain of rats were detected and a significantly higher red fluorescence signal was emitted from PDLSC-treated rats. It was more around the periphery of the ischemic area than in non-infarcted areas on the contralateral side. In vivo, PDLSCs can pass from the blood–brain barrier more efficiently and migrate to ischemic areas faster than DPSCs. The size of the infarcted area was significantly smaller in PDLSCs treated group. This finding indicates that the neural differentiation potential and in vivo therapeutic effect of PDLSCs are more evident than DPSCs [61].

The conditioned medium of PDLSCs can reduce inflammatory damage in the animal model of MS [62]. Also, purified extracellular vesicles from PDLSCs have a similar effect. A mixed population of shedding vesicles and exosomes which contain anti‐inflammatory cytokines TGF‐β and IL‐10 stained positive for surface mesenchymal antigens CD29 and CD90. Moreover, the secretome content of MSCs as a reservoir of regenerating neuronal factors is a key regulator of the neurogenic niche [62, 63].

Conditioned medium (CM) of the cells is useful in enhancing long-term neuronal regeneration in spinal cord injury [64]. The injection of vesicles or PDLSCs conditioned medium (PDLSCs-CM) of patients with relapsing‐remitting MS to mouse model of MS showed the same result; the functional and pathology‐independent ability of PDLSCs niche was demonstrated that can be applied for several other inflammatory and neurodegenerative diseases. PDLSCs-CM obtained from MS patients under hypoxic conditions when injected (1300 μG of hPDLSCs-CM/mouse) in mouse with autoimmune encephalomyelitis (EAE) as MS animal model leads to reduced pro-inflammatory and induced anti-inflammatory cytokines [62]. It can potentially modulate markers of oxidative stress, autophagy and apoptosis through increasing the expression of beclin‐1 and LC3 (principal markers of autophagy) and reducing the interferon‐γ and IL‐17 expression (65). PDLSC secretome can reduce oxidative stress and inflammation in injured neurons and also can increase the functionality of the PI3K/Akt/mTOR axis which results in restoring BDNF production. Moreover, the conditioned medium has a neuroprotective effect due to containing NT-3, and IL‐10, and also the presence of growth factors and immunomodulatory cytokines [66].

In an in vitro study, the immunosuppressive, neuroprotective and anti-apoptotic properties of hPDLSCs-CM obtained from patients with relapsing remitting- MS have been demonstrated. The PDLSC-CM contained extracellular vesicles with anti-inflammatory cytokines (TGF-β and IL-10). Preconditioning of lipopolysaccharide (LPS)-stimulated NSC34 mouse motoneurons with the PDLSCs-CM led to the enhanced level of NFκB and TLR4 and decreased amount of IκB-α in inflammation-exposed motoneurons. Moreover, neuroprotective markers such as nestin, NGF, NFL 70, GAP-43 as well as inflammatory cytokines and apoptotic markers were modulated in this study [67].

In the eye, the neuroprotective factors of hPDLSCs have beneficial effects on degenerated retina and photoreceptor survival, possibly by an anti-apoptotic mechanism. Experiments confirmed that PDLSCs alone exhibited neuroprotective effect and enhanced neurite regeneration in retinal tissue without macrophage recruitment. In in vitro experiments, the neuroprotection of PDLSCs can be increased when they are in direct cell–cell with retinal ganglion cells (RGCs) in retinal explant culture. This process increased BDNF secretion by twofold, but the mechanism of this positive feedback needs further investigation [68, 69].

PDLSCs are capable of cell replacement and differentiation into the functional RGCs [12, 54]. The intravitreal transplantation of PDLSCs can promote survival of RGC and regeneration of axons in a rat model of optic nerve injury which implies a potential therapy for neurological diseases via neuroprotection [70].

The differentiated PDLSCs to RGCs expressed markers related to neurons and RGCs including β-III tubulin, POU4F2, ATOH7, MAP2, TAU, SIX3 and NEUROD1 and have the ability to form synapses. They showed spontaneous electrical activities as well as glutamate-induced calcium responses which are the characteristics of functional neurons. During the induction process, the expressions of VEGF and PTEN as miRNA-targeted candidates were significantly upregulated [69].

Moreover, the PDLSCs can be induced to retinal fate expressing photoreceptor makers [68]. PDLSCs produced neurospheres in low attachment culture and express neural progenitor markers. On a surface with Matrigel coating, neurospheres showed rosette-like outgrowth and expressed eye-related factors; 94% of the cells tested positive for Pax6 (retinal progenitor marker). They produced markers of photoreceptors such as rhodopsin, and its kinase and Nrl; also, they showed considerable excitatory glutamate response after continuous induction. Induction into retinal fate was carried out by a protocol to development of retinogenesis and anterior neural plate through inhibition of BMP and Wnt signaling [68, 71].

The PDLSCs have intrinsic PAX6 expression which is mostly located inside of the cytoplasm. After induction to retinal fate, in the majority of cells Pax6 protein was translocated into the nucleus. Pax6 nucleocytoplasmic translocation and also its nuclear retention are mediated by TGFβ signaling and interaction of Smad3-SPARC [72–74]. However, it is not yet to be determined whether the reason of Pax6 translocation is the changes of the retinogenesis signaling or is due to other factors.

## Peripheral nerve system treatment

**Fig. 4:**
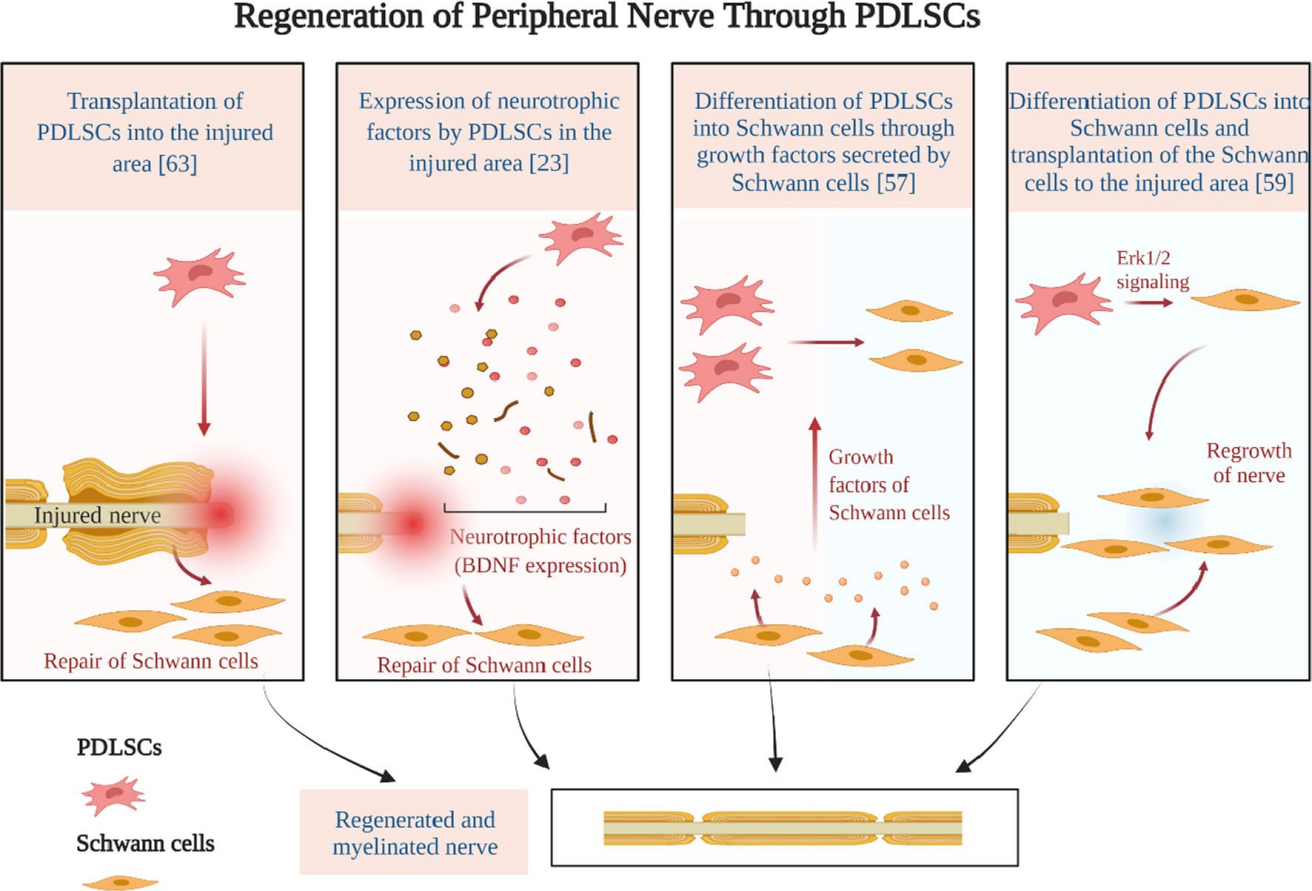
Repair and regeneration of the peripheral nerve injury by different mechanisms through periodontal ligament stem cells. Created with BioRender.com

PDLSCs through the expression of several neural growth and differentiation factors have shown important neurotrophic effects in peripheral nerve injury [41] (Fig. 4). A promising strategy for peripheral nerve repair is Schwann cell (SC) transplantation. PDLSCs are one of the cell sources that can be differentiated into SC-like cells [50, 75]. Different pathways are involved in this process, but it is demonstrated that Erk1/2 signaling is one of the most important pathways in the differentiation process of PDLSCs to SCs. During this process, Erk1/2 signaling inhibition leads to reduced expression of SCs specific markers such as P75, S100 and GFAP as well as decreased myelinization-related genes including P0, krox-20 and Oct-6 [76–78].

Reports demonstrated that indirect co-culturing of heterogenous Schwann cells and PDLSCs can induce PDLSCs toward Schwann cell phenotype through allogenic neurotrophic factors released by Schwann cells. Neural differentiation began 24 h after co-culture, and the most detectable neural differentiation of PDLSCs appeared 14 days later [75].

In a study, the biological behavior of induced PDLSCs into Schwann cells (SC) was investigated on the sandblasted and acid-etched titanium surface. The goal of this study was to seek whether the SC-like cells placed on the implant can replace SCs in injured nerve endings. They used PDLSCs instead of SCs due to difficulties obtaining pure and ample sources of SCs. The study showed that the differentiated PDLSCs had the highest expression of SC markers and proteins on the SA titanium surface [79].

Furthermore, SH-SY5Y cells of human neuroblastoma were incubated with CM of the SCAPs, PDLSCs and DPSCs. Neurotrophic factors in the conditioned medium of these stem cells can induce differentiation of neuroblastoma SH-SY5Y cells to cells with neurites outgrowth. Stimulation of these cells with a combination of growth factors leads to a significant upregulation of GDNF and BDNF in the CM of SCAPs and DPSCs but not in the PDLSCs. Their CM leads to an increase in the length of the neurite and also the number of cells producing neurites. BDNF in their CM plays a vital role in the stimulation of neurite outgrowth [41, 80]. In in vivo, conduits seeded with SCAPs, DPSCs, PDLSCs and rat Schwann cells (rSC) were utilized to treat a 10-mm nerve defect in a rat model of sciatic nerve injury. The greatest distance of axon regeneration was created in rSC and SCAP-treated group. All seeded conduits significantly reduced the expression of caspase-3 in injured area compared to group with empty conduit. In vivo assay detected BDNF expression in the area of the injected cells. All of these stem cells significantly increased axon regeneration and led to neuroprotective effects in neurons of the dorsal root ganglia [41]. The ability of these three stem cells in combination with a fibrin glue conduit showed that SCAP and PDLSCs have neural repair potential through direct differentiation toward neural cells or they participate in nerve regeneration through paracrine manner [70, 80, 81]. A higher level of neuronal markers was detected in PDLSCs and SCAP compared to DPSCs based on mRNA levels and immunocytochemical staining [82].

Moreover, PDLSCs were transplanted into rats with the crush injury in left mental nerve. Concurrently, control group received autologous SCs and PBS. After 5 days of injection, expression of NGF receptor was significantly increased, and after 2 and 4 weeks, the sensory function of the cells was significantly recovered. Also, the numbers of myelinated axons and sensory neurons were significantly amplified. These results demonstrated that the effectiveness of PDLSCs injection for axonal regeneration was equivalent to reparative ability of Schwann cells. Moreover, human PDLSCs through differentiation into Schwann cells can be alternative source for the autologous SCs in order for the regeneration of the peripheral nerve injury. [80, 81].

## Main challenges and future perspectives

Periodontal ligament stem cells can be an appealing source of stem cells for regenerative medicine, including nerve regeneration. Using PDLSCs presents a difficulty in terms of isolation and culturing approaches. It is necessary to investigate the unique culturing procedure required for neuronal differentiation. These cells are heterogeneous sources with various differentiation priorities for multiple cell fates; identifying the precise subgroup of these cells could be a significant step. It is necessary to develop the most efficient methodology for obtaining and storing PDLSC-conditioned media containing various neurotrophic and anti-inflammatory factors. Based on their neurogenic potential, the protein content of the conditioned media of these stem cells has to be investigated further. The effect of their exosome on neural differentiation has yet to be determined. Although significant in vitro and in vivo investigations were carried out in order for neural differentiation of these cells into diverse types of neurons, more preclinical and clinical studies are needed to elucidate their therapeutic potential for neurodegenerative diseases.

## Conclusion

Periodontal ligament stem cells have favorable biological features that can be obtained from the periodontal ligament as an autologous source. They are readily available, stable, and sufficient sources of cells that do not trigger immunological responses and have great features such as immune modulation and neuroprotection. PDLSCs are a unique type of cell that can be used in repairing neural tissue since they are originated from neural crest. They have potential to differentiate into various cells such as neural-like cells under inductive conditions. Various compounds in their conditioned media and extracellular vesicles can replace cells in most tasks, but it needs further research into their potential usage. PDLSCs can release neurotrophic factors which can help in the myelination and the regrowth of neurons. In vitro and in vivo investigations have reported that they are useful in treating central and peripheral nervous system diseases. Furthermore, when paired with biomaterials, PDLSCs could have significant implications for neural tissue repair, which has to be investigated further. To summarize, these cells have a good potential for use in clinical investigations for neural regeneration, but there are still a number of challenges to conquer.

## Data Availability

Not applicable.

## Abbreviations

PDLSCs: Periodontal ligament stem cells
MSCs: Mesenchymal stem cells
BBB: Blood–brain barrier
GMSCs: Gingival MSCs
DFSC: Dental follicle stem cells
SCAP: Stem cells from apical papilla
DPSCs: Dental pulp stem cells
SHED: Stem cells from exfoliated deciduous teeth
CNCCs: Cranial neural crest-derived ectomesenchymal cells
PDL: Periodontal ligament
AD: Alzheimer’s disease
SCI: Spinal cord injury
hBMGCs: Human bone marrow mesenchymal stem cells
bFGF: Basic fibroblast growth factor
IGF-1: Insulin-like growth factor-1
NGF: Nerve growth factor
EGF: Epidermal growth factor
NCSCs: Neural crest stem cells
OA: Okadaic acid
MS: Multiple sclerosis
CM: Conditioned medium
PDLSCs-CM: PDLSCs conditioned medium
EAE: Autoimmune encephalomyelitis
LPS: Lipopolysaccharide
RGCs: Retinal ganglion cells
SC: Schwann cell
rSC: Rat Schwann cells

## References

[1] Gutiérrez-Gutiérrez G, Sereno M, Miralles A, Casado-Sáenz E, Gutiérrez-Rivas E (2010). Chemotherapy-induced peripheral neuropathy: clinical features, diagnosis, prevention and treatment strategies. Clin Transl Oncol.

[2] Fortino VR, Chen RS, Pelaez D, Cheung HS (2014). Neurogenesis of neural crest-derived periodontal ligament stem cells by EGF and bFGF. J Cell Physiol.

[3] Mueller BK, Mueller R, Schoemaker H (2009). Stimulating neuroregeneration as a therapeutic drug approach for traumatic brain injury. Br J Pharmacol.

[4] Nagappan PG, Chen H, Wang D-Y (2020). Neuroregeneration and plasticity: a review of the physiological mechanisms for achieving functional recovery postinjury. Mil Med Res.

[5] Chen J, Chopp M (2006). Neurorestorative treatment of stroke: cell and pharmacological approaches. NeuroRx.

[6] Grunwell J, Illes J, Karkazis K (2009). Advancing neuroregenerative medicine: a call for expanded collaboration between scientists and ethicists. Neuroethics.

[7] Xiao L, Nasu M (2014). From regenerative dentistry to regenerative medicine: progress, challenges, and potential applications of oral stem cells. Stem Cells Cloning: Adv Appl.

[8] Sandu N, Momen-Heravi F, Sadr-Eshkevari P, Schaller B (2012). Molecular imaging for stem cell transplantation in neuroregenerative medicine. Neurodegener Dis.

[9] Ng TK, Fortino VR, Pelaez D, Cheung HS (2014). Progress of mesenchymal stem cell therapy for neural and retinal diseases. World J Stem Cells.

[10] Cooney DS, Wimmers EG, Ibrahim Z, Grahammer J, Christensen JM, Brat GA (2016). Mesenchymal stem cells enhance nerve regeneration in a rat sciatic nerve repair and hindlimb transplant model. Sci Rep.

[11] Schmidt A, Ladage D, Steingen C, Brixius K, Schinköthe T, Klinz F-J (2006). Mesenchymal stem cells transmigrate over the endothelial barrier. Eur J Cell Biol.

[12] Steingen C, Brenig F, Baumgartner L, Schmidt J, Schmidt A, Bloch W (2008). Characterization of key mechanisms in transmigration and invasion of mesenchymal stem cells. J Mol Cell Cardiol.

[13] Galindo LT, Filippo TR, Semedo P, Ariza CB, Moreira CM, Camara NO (2011). Mesenchymal stem cell therapy modulates the inflammatory response in experimental traumatic brain injury. Neurol Res Int.

[14] Volponi A, Sharpe P (2013). The tooth–a treasure chest of stem cells. Br Dent J.

[15] Andrukhov CB, Blufstein A, Rausch-Fan X (2019). Immunomodulatory properties of dental tissue-derived mesenchymal stem cells: implication in disease and tissue regeneration. World J Stem Cells.

[16] Li D, Zou X-Y, El-Ayachi I, Romero LO, Yu Z, Iglesias-Linares A (2019). Human dental pulp stem cells and gingival mesenchymal stem cells display action potential capacity in vitro after neuronogenic differentiation. Stem cell Rev Rep.

[17] Lin NH, Gronthos S, Bartold P (2008). Stem cells and periodontal regeneration. Aust Dent J.

[18] Ng TK, Huang L, Cao D, Yip YW-Y, Tsang WM, Yam GH-F (2015). Cigarette smoking hinders human periodontal ligament-derived stem cell proliferation, migration and differentiation potentials. Sci Rep.

[19] Coura G, Garcez R, De Aguiar CM, Alvarez-Silva M, Magini R, Trentin A (2008). Human periodontal ligament: a niche of neural crest stem cells. J Periodontal Res.

[20] Huang CC, Pelaez D, Bendala JD, Garcia-Godoy F, Cheung HS (2009). Plasticity of stem cells derived from adult periodontal ligament. Regen Med.

[21] Song JS, Kim S-O, Kim S-H, Choi H-J, Son H-K, Jung H-S (2012). In vitro and in vivo characteristics of stem cells derived from the periodontal ligament of human deciduous and permanent teeth. Tissue Eng Part A.

[22] Iwata T, Yamato M, Zhang Z, Mukobata S, Washio K, Ando T (2010). Validation of human periodontal ligament-derived cells as a reliable source for cytotherapeutic use. J Clin Periodontol.

[23] Washio K, Iwata T, Mizutani M, Ando T, Yamato M, Okano T (2010). Assessment of cell sheets derived from human periodontal ligament cells: a pre-clinical study. Cell Tissue Res.

[24] Yamaza T, Kentaro A, Chen C, Liu Y, Shi Y, Gronthos S (2010). Immunomodulatory properties of stem cells from human exfoliated deciduous teeth. Stem Cell Res Ther.

[25] Zhang J, An Y, Gao L-N, Zhang Y-J, Jin Y, Chen F-M (2012). The effect of aging on the pluripotential capacity and regenerative potential of human periodontal ligament stem cells. Biomaterials.

[26] Seo B, Miura M, Gronthos S (2004). Erratum: investigation of multipotent postnatal stem cells from human periodontal ligament ligament (Lancet (2004) 364 149-155). Lancet.

[27] Trubiani O, Di Primio R, Traini T, Pizzicannella J, Scarano A, Piattelli A (2005). Morphological and cytofluorimetric analysis of adult mesenchymal stem cells expanded ex vivo from periodontal ligament. Int J Immunopathol Pharmacol.

[28] Trubiani O, Piattelli A, Gatta V, Marchisio M, Diomede F, D'Aurora M (2015). Assessment of an efficient xeno-free culture system of human periodontal ligament stem cells. Tissue Eng Part C Methods.

[29] Trubiani O, Diomede F (2014). Xeno-free culture of human periodontal ligament stem cells.

[30] Iriarte CGT, Ramírez OR, García AM, Terán SLV, Clavel JFG (2017). Isolation of periodontal ligament stem cells from extracted premolars. Simpl Method Rev Odontol Mex.

[31] Chopra H, Liao C, Zhang C, Pow E (2018). Lapine periodontal ligament stem cells for musculoskeletal research in preclinical animal trials. J Transl Med.

[32] Banavar SR, Rawal SY, Paterson IC, Singh G, Davamani F, Khoo SP (2021). Establishing a technique for isolation and characterization of human periodontal ligament derived mesenchymal stem cells. Saudi Dent J.

[33] Mohebichamkhorami F, Niknam Z, Khoramjouy M, Heidarli E, Ghasemi R, Hosseinzadeh S (2022). Brain homogenate of rat model of alzheimer disease modifies secretome of 3D cultured periodontal ligament stem cells a potential neuroregenerative therapy. Iran J Pharm Res.

[34] Park JC, Kim JM, Jung IH, Kim JC, Choi SH, Cho KS (2011). Isolation and characterization of human periodontal ligament (PDL) stem cells (PDLSCs) from the inflamed PDL tissue: in vitro and in vivo evaluations. J Clin Periodontol.

[35] Tomokiyo A, Yoshida S, Hamano S, Hasegawa D, Sugii H, Maeda H (2018). Detection, characterization, and clinical application of mesenchymal stem cells in periodontal ligament tissue. Stem Cells Int.

[36] Ballerini P, Diomede F, Petragnani N, Cicchitti S, Merciaro I, Cavalcanti MF (2017). Conditioned medium from relapsing-remitting multiple sclerosis patients reduces the expression and release of inflammatory cytokines induced by LPS-gingivalis in THP-1 and MO3. 13 cell lines. Cytokine.

[37] Morsczeck C, Götz W, Schierholz J, Zeilhofer F, Kühn U, Möhl C (2005). Isolation of precursor cells (PCs) from human dental follicle of wisdom teeth. Matrix Biol.

[38] Le Douarin N, LeDouarin NM, Kalcheim C (1999). The neural crest.

[39] Sakai K, Yamamoto A, Matsubara K, Nakamura S, Naruse M, Yamagata M (2012). Human dental pulp-derived stem cells promote locomotor recovery after complete transection of the rat spinal cord by multiple neuro-regenerative mechanisms. J Clin Investig.

[40] Raza SS, Wagner AP, Hussain YS, Khan MA (2018). Mechanisms underlying dental-derived stem cell-mediated neurorestoration in neurodegenerative disorders. Stem Cell Res Ther.

[41] Kolar MK, Itte VN, Kingham PJ, Novikov LN, Wiberg M, Kelk P (2017). The neurotrophic effects of different human dental mesenchymal stem cells. Sci Rep.

[42] Yamamoto T, Osako Y, Ito M, Murakami M, Hayashi Y, Horibe H (2016). Trophic effects of dental pulp stem cells on Schwann cells in peripheral nerve regeneration. Cell Transplant.

[43] do Couto Nisssscola F, Marques MR, Odorcyk F, Arcego DM, Petenuzzo L, Aristimunha D (2017). Neuroprotector effect of stem cells from human exfoliated deciduous teeth transplanted after traumatic spinal cord injury involves inhibition of early neuronal apoptosis. Brain Res.

[44] Yang C, Li X, Sun L, Guo W, Tian W (2017). Potential of human dental stem cells in repairing the complete transection of rat spinal cord. J Neural Eng.

[45] Widera D, Grimm W-D, Moebius JM, Mikenberg I, Piechaczek C, Gassmann G (2007). Highly efficient neural differentiation of human somatic stem cells, isolated by minimally invasive periodontal surgery. Stem Cells Dev.

[46] Martens W, Bronckaers A, Politis C, Jacobs R, Lambrichts I (2013). Dental stem cells and their promising role in neural regeneration: an update. Clin Oral Invest.

[47] Bueno C, Martínez-Morga M, Martínez S (2019). Non-proliferative neurogenesis in human periodontal ligament stem cells. Sci Rep.

[48] Bueno C, Martínez-Morga M, García-Bernal D, Moraleda JM, Martínez S (2021). Differentiation of human adult-derived stem cells towards a neural lineage involves a dedifferentiation event prior to differentiation to neural phenotypes. Sci Rep.

[49] Li X, Liao D, Sun G, Chu H (2020). Odontogenesis and neuronal differentiation characteristics of periodontal ligament stem cells from beagle dog. J Cell Mol Med.

[50] Li X, Gong P, Liao D (2010). In vitro neural/glial differentiation potential of periodontal ligament stem cells. Arch Med Sci: AMS.

[51] Ng TK, Yang Q, Fortino VR, Lai NYK, Carballosa CM, Greenberg JM (2019). MicroRNA-132 directs human periodontal ligament-derived neural crest stem cell neural differentiation. J Tissue Eng Regen Med.

[52] Maeda A, Kobayashi Y, Saito T, Togitani K, Kawahigashi N, Tanosaki R (1999). Central nervous system relapse with multiple brain masses in an acute promyelocytic leukemia patient treated with all-trans retinoic acid. [Rinsho ketsueki] Jpn J Clin Hematol.

[53] Dent EW, Meiri KF (1998). Distribution of phosphorylated GAP-43 (neuromodulin) in growth cones directly reflects growth cone behavior. J Neurobiol.

[54] Korshunova I, Novitskaya V, Kiryushko D, Pedersen N, Kolkova K, Kropotova E (2007). GAP-43 regulates NCAM-180-mediated neurite outgrowth. J Neurochem.

[55] Diomede F, Zini N, Pizzicannella J, Merciaro I, Pizzicannella G, D’Orazio M (2018). 5-Aza exposure improves reprogramming process through embryoid body formation in human gingival stem cells. Front Genet.

[56] Trubiani O, Guarnieri S, Diomede F, Mariggiò MA, Merciaro I, Morabito C (2016). Nuclear translocation of PKCα isoenzyme is involved in neurogenic commitment of human neural crest-derived periodontal ligament stem cells. Cell Signal.

[57] Ansari S, Diniz IM, Chen C, Sarrion P, Tamayol A, Wu BM (2017). Human periodontal ligament-and gingiva-derived mesenchymal stem cells promote nerve regeneration when encapsulated in alginate/hyaluronic acid 3D scaffold. Adv Healthc Mater.

[58] Bueno C, Ramirez C, Rodríguez-Lozano FJ, Tabarés-Seisdedos R, Rodenas M, Moraleda JM (2013). Human adult periodontal ligament-derived cells integrate and differentiate after implantation into the adult mammalian brain. Cell Transplant.

[59] Wang D, Wang Y, Tian W, Pan J (2019). Advances of tooth-derived stem cells in neural diseases treatments and nerve tissue regeneration. Cell Prolif.

[60] Trubiani O, Giacoppo S, Ballerini P, Diomede F, Piattelli A, Bramanti P (2016). Alternative source of stem cells derived from human periodontal ligament: a new treatment for experimental autoimmune encephalomyelitis. Stem Cell Res Ther.

[61] Wu T, Xu W, Chen H, Li S, Dou R, Shen H (2020). Comparison of the differentiation of dental pulp stem cells and periodontal ligament stem cells into neuron-like cells and their effects on focal cerebral ischemia. Acta Biochim Biophys Sin.

[62] Rajan TS, Giacoppo S, Diomede F, Ballerini P, Paolantonio M, Marchisio M (2016). The secretome of periodontal ligament stem cells from MS patients protects against EAE. Sci Rep.

[63] Yeasmin S, Ceccarelli J, Vigen M, Carrion B, Putnam AJ, Tarle SA (2014). Stem cells derived from tooth periodontal ligament enhance functional angiogenesis by endothelial cells. Tissue Eng Part A.

[64] Man RC, Sulaiman N, Idrus RBH, Ariffin SHZ, Wahab RMA, Yazid MD (2019). Insights into the effects of the dental stem cell secretome on nerve regeneration: towards cell-free treatment. Stem Cells Int.

[65] Giacoppo S, Thangavelu SR, Diomede F, Bramanti P, Conti P, Trubiani O (2017). Anti-inflammatory effects of hypoxia-preconditioned human periodontal ligament cell secretome in an experimental model of multiple sclerosis: a key role of IL-37. FASEB J.

[66] Pizzicannella J, Gugliandolo A, Orsini T, Fontana A, Ventrella A, Mazzon E (2019). Engineered extracellular vesicles from human periodontal-ligament stem cells increase VEGF/VEGFR2 expression during bone regeneration. Front Physiol.

[67] Rajan TS, Giacoppo S, Trubiani O, Diomede F, Piattelli A, Bramanti P (2016). Conditioned medium of periodontal ligament mesenchymal stem cells exert anti-inflammatory effects in lipopolysaccharide-activated mouse motoneurons. Exp Cell Res.

[68] Huang L, Liang J, Geng Y, Tsang W-M, Yao X, Jhanji V (2013). Directing adult human periodontal ligament–derived stem cells to retinal fate. Invest Ophthalmol Vis Sci.

[69] Ng TK, Yung JS, Choy KW, Cao D, Leung CK, Cheung HS (2015). Transdifferentiation of periodontal ligament-derived stem cells into retinal ganglion-like cells and its microRNA signature. Sci Rep.

[70] Cen LP, Ng TK, Liang JJ, Zhuang X, Yao X, Yam GHF (2018). Human periodontal ligament-derived stem cells promote retinal ganglion cell survival and axon regeneration after optic nerve injury. Stem Cells.

[71] Kadar K, Kiraly M, Porcsalmy B, Molnar B, Racz G, Blazsek J (2009). Differentiation potential of stem cells from human dental origin-promise for tissue engineering. J Physiol Pharmacol.

[72] Grocott T, Frost V, Maillard M, Johansen T, Wheeler GN, Dawes LJ (2007). The MH1 domain of Smad3 interacts with Pax6 and represses autoregulation of the Pax6 P1 promoter. Nucleic Acids Res.

[73] Mu Y, Gudey SK, Landström M (2012). Non-Smad signaling pathways. Cell Tissue Res.

[74] Shubham K, Mishra R (2012). Pax6 interacts with SPARC and TGF-β in murine eyes. Mol Vis.

[75] Li X, Liao D, Gong P, Tang H (2009). Neural differentiation of periodontal ligament stem cells induced by neurotrophic Schwann cells factors. Arch Med Sci.

[76] Trubiani O, Pizzicannella J, Caputi S, Marchisio M, Mazzon E, Paganelli R (2019). Periodontal ligament stem cells: current knowledge and future perspectives. Stem cells and development.

[77] Dapeng L, Xiaojie L, Ping G, Yan D, Gang S (2014). Erk1/2 signalling is involved in the differentiation of periodontal ligament stem cells to Schwann cells in dog. Arch Oral Biol.

[78] Soundararajan P, Fawcett JP, Rafuse VF (2010). Guidance of postural motoneurons requires MAPK/ERK signaling downstream of fibroblast growth factor receptor 1. J Neurosci.

[79] Xiaojie L, Dapeng L, Ping G, Yan D, Gang S (2014). Biological behavior of neurally differentiated periodontal ligament stem cells on different titanium implant surfaces. J Biomed Mater Res Part A.

[80] Pisciotta A, Bertoni L, Vallarola A, Bertani G, Mecugni D, Carnevale G (2020). Neural crest derived stem cells from dental pulp and tooth-associated stem cells for peripheral nerve regeneration. Neural Regen Res.

[81] Li B, Jung H-J, Kim S-M, Kim M-J, Jahng JW, Lee J-H (2013). Human periodontal ligament stem cells repair mental nerve injury. Neural Regen Res.

[82] Lee J-H, Um S, Song I-S, Kim HY, Seo BM (2014). Neurogenic differentiation of human dental stem cells in vitro. J Korean Assoc Oral Maxillofac Surg.

